# Systematic review of cholangiocarcinoma in Africa: epidemiology, management, and clinical outcomes

**DOI:** 10.1186/s12876-023-02687-6

**Published:** 2023-03-11

**Authors:** Akwi W. Asombang, Nathaniel Chishinga, Mouhand F. Mohamed, Alick Nkhoma, Jackson Chipaila, Bright Nsokolo, Martha Manda-Mapalo, Joao Filipe G. Montiero, Lewis Banda, Kulwinder S. Dua

**Affiliations:** 1 Division of Gastroenterology/Hepatology, Massachusetts General Hospital, 15 Parkman Street, Wang 5, Boston, MA 02114 USA; 2 Department of Internal Medicine, Piedmont Athens Regional Hospital, 1270 Prince Avenue, Suite 102, Athens, GA 30606 USA; 3 Department of Medicine, Warren Alpert Medical School of Brown University, Providence, RI 02903 USA; 4 Department of Gastroenterology, Royal Stoke University Hospital, University Hospitals of North Midlands NHS Trust, Staffordshire, ST4 6QG UK; 5 Department of Surgery, University Teaching Hospital-Adult Hospital, Lusaka, 10101 Zambia; 6 Department of Internal Medicine, Levy Mwanawasa Medical University, Levy Mwanawasa University Teaching Hospital, Lusaka, 10101 Zambia; 7 Division of Hematology/Oncology, Department of Internal Medicine, The University of New Mexico, Albuquerque, NM 87106 USA; 8 Department of Medicine, Brown University, Providence, RI 02903 USA; 9 Hematology/Oncology, Cancer Disease Hospital, Lusaka, 10101 Zambia; 10 Department of Medicine and Pediatrics, Medical College of Wisconsin, Milwaukee, WI 53226 USA

**Keywords:** Cholangiocarcinoma, Epidemiology, Risk factors, Systematic review, Africa

## Abstract

**Background:**

The prevalence, management, and clinical outcomes of cholangiocarcinoma in Africa are unknown. The aim is to conduct a comprehensive systematic review on the epidemiology, management, and outcomes of cholangiocarcinoma in Africa.

**Methods:**

We searched PubMed, EMBASE, Web of Science and CINHAL from inception up to November 2019 for studies on cholangiocarcinoma in Africa. The results reported follow PRISMA guidelines. Quality of studies and risk of bias were adapted from a standard quality assessment tool. Descriptive data were expressed as numbers with proportions and Chi-squared test was used to compare proportions. *P* values < 0.05 were considered significant.

**Results:**

A total of 201 citations were identified from the four databases. After excluding duplicates, 133 full texts were reviewed for eligibility, and 11 studies were included. The 11 studies are reported from 4 countries only: 8 are from North Africa (Egypt 6 and Tunisia 2), and 3 in Sub-Saharan Africa (2 in South Africa, 1 in Nigeria). Ten studies reported management and outcomes, while one study reported epidemiology and risk factors. Median age for cholangiocarcinoma ranged between 52 and 61 years. Despite the proportion with cholangiocarcinoma being higher among males than females in Egypt, this gender disparity could not be demonstrated in other African countries. Chemotherapy is mainly used for palliative care. Surgical interventions are curative and prevent cancer progression. Statistical analyses were performed with Stata 15.1.

**Conclusion:**

The known global major risk factors such as primary sclerosing cholangitis, *Clonorchis sinensis* and *Opisthorchis viverrini* infestation are rare. Chemotherapy treatment was mainly used for palliative treatment and was reported in three studies. Surgical intervention was described in at least 6 studies as a curative modality of treatment. Diagnostic capabilities such as radiographic imaging and endoscopic are lacking across the continent which most likely plays a role in accurate diagnosis.

## Background

Cholangiocarcinoma (CCA) is a rare and aggressive cancer that arises from the epithelium of the intrahepatic or the extrahepatic bile ducts. CCA can also arise from hepatic progenitor cells [1]. Globally, the incidence of cholangiocarcinoma varies from as high as 113 per 100,000 in Northern Thailand, 5.7/100,000 in Southern Thailand, 2.2/100,000 in UK, 1.1/100,000 in USA and 0.3/100,000 in Israel [2]. In both males and females, the incidence rises at age 60 to 70 years, rarely diagnosed before age 40 years, with a higher incidence and mortality in men compared to women [2–4]. Little is known of CCA in Africa. Patients with CCA can present with jaundice pruritus, acholic stool and steatorrhea depending on tumor location and stage of presentation, but they can also present with abdominal pain and weight loss. Some patients have their CCAs detected as incidentalomas during cross sectional imaging for abdominal symptoms, or during hepatoma screening in those with underlying cirrhosis. Screening programs exist for detection of gall bladder or biliary cholangiocarcinoma in patients with underlying primary sclerosing cholangitis (PSC) using MRCP and CA19-9 [5]. The management of CCA is dependent on anatomical location of tumor, staging and histology [6].

Anatomically CCA are classified as: Intrahepatic cholangiocarcinoma (iCCA) if the tumor is located proximal to the secondary branches of the right and left hepatic ducts, perihilar cholangiocarcinoma (pCCA) if located between the secondary branches of the right and left hepatic ducts and the common hepatic duct proximal to the origin of the cystic duct, and distal cholangiocarcinoma (dCCA) if involving the common bile duct but not the ampulla of Vater [2, 7]. Surgical resection is curative in early iCCA and if R0 resection is achieved, can give up to 36 months disease specific survival. There is a risk of recurrence in up to 62.2% of patients [8]. Surgical resection is also curative in dCCA early stage disease for R0 resection which entails a Whipple’s procedure, although the risk of recurrence is high with 5-year survival of 27% [9]. Surgical resection is the treatment of choice for early stage Bismuth-Corlette pCCA, although liver transplantation is emerging as the preferred treatment option with up to 76% 5-year survival with neoadjuvant chemoradiotherapy [10]. Neoadjuvant and adjuvant chemotherapy does improve survival in all types of CCA. Hyperbilirubinemia due to biliary obstruction results in a pro-inflammatory state that negatively impacts the post-operative outcomes [11]. Hyperbilirubinemia due to biliary obstruction results in a pro-inflammatory state that negatively impacts the post-operative outcomes [11]. Endoscopic Retrograde Cholangiopancreatography (ERCP) and percutaneous transhepatic biliary drainage (PTBD) are the procedures most commonly used for biliary drainage; in both the curative (preoperative) and palliative setting. PTBD is considered to be more advantageous in the preoperative setting, for better priming for surgery, and there is a lower risk of postprocedural complications such as cholangitis. In the palliative setting, either technique can be used depending on the expertise of the operators, however in cases where the bilirubin is very high, the stenosis is lengthy, or in the presence of cholangitis, ERCP failure, or altered biliary anatomy—PTBD is recommended over ERCP [12].

Many patients present with unresectable or metastatic disease; as such the median survival rate is as low as 3 to 6 months in the USA [13].

Risk factors for developing CCA include occupation, which can be due to chemical exposure, chronic biliary inflammation, chronic hepatic inflammation, and cirrhosis of any cause and congenital and acquired causes of cholestasis leading to biliary inflammation. Nonetheless, risk factors are not identifiable in 50% of cases [2]. CCA has been documented in workers at printing companies in Japan exposed to high concentrations of 1,2-dichloropropane (1,2-DCP) and dichloromethane [14]. CCA has been shown to develop decades after administration of the radiologic contrast medium thorotrast, that was used for cerebral angiography in the 1950s [15]. Autoimmune conditions, especially PSC have been associated with increased risk of cholangiocarcinoma in elderly patients in the USA [16]. Heavy infestation by liver flukes *Clonorchis sinensis* and *Opisthorchis viverrini* due to eating raw fish is a known cause of CCA in Asian countries where these flukes are endemic, including Korea, China, Taiwan, and Vietnam [17]. Other liver flukes *Fasciola hepatica* and *Fasciola gigantica,* cause fascioliasis in sheep and cattle, and can infest other herbivores, are a known zoonotic cause of human fasciolasis from eating water cress and drinking water with Lymnaeidae snails, that are intermediate hosts, can cause chronic cholestasis [18]. Unlike the *Clonorchis sinensis* and *Opisthorchis viverrini*, there is no direct causal like between *Fasciola hepatica* and *Fasciola gigantica,* to CCA [18]. Chronic intrahepatic cholestasis due to pigment stones can occur to due chronic hemolysis associated with hemolytic conditions such as sickle cell disease, thalassemia and red blood cell enzyme disorders such as G6PD deficiency. Although hepatolithiasis is a known risk factor for CCA, a direct link between hemolytic anemia and CCA has not been established [19]. Bacterial infections with *Helicobacter* species causing chronic cholangitis and cholecystitis have been implicated in the etiological role in biliary cancers [20] Obesity has been associated with the increased risk of cholangiocarcinoma [21]. The use of GLP-1 analogues, used in treatment of diabetes is associated with an increased risk of bile duct and gallbladder disease requiring hospital admission including cholelithiasis, cholecystitis, cholangitis. Over the long term as inflammation and cholestasis are associated with CCA, there is a possibility that use of these medications could pose a risk to development of CCA [22]. In a recent review of risk factors for CCA in Eastern and Western countries, choledochal cysts, cirrhosis, choledocholithiasis, hepatitis B virus infection have been found to be associated with CCA [2].

To date, no systematic review or meta-analysis of studies on CCA in Africa has been conducted. We address this gap by conducting a comprehensive systematic review on the epidemiology, management, and outcomes of CCA in Africa.

## Materials and methods

### Search strategy and study identification

We searched PubMed, EMBASE, Web of Science, and CINAHL from inception to November 2019 for primary publications. Searches were limited to human studies conducted in Africa and to English-language publications. The following search terms were used in the four databases: (Cholangiocar* OR cholangio cancer OR cholangio tumo*) AND (epidemiology OR incidence OR prevalence OR risk factor* OR treatment OR management OR outcome*) AND (Africa OR East* Africa OR West Africa OR Southern Africa OR North* Africa OR Algeria* OR Angola* OR Benin OR Botswana OR “Burkina Faso” OR Burundi OR Cameroon* OR "Cabo Verde" OR “Cape Verde” OR “Central African Republic” OR Chad OR Comoros OR Congo OR “Cote d`Ivoire” OR Djibouti OR Egypt* OR “Democratic Republic of Congo” OR “Equatorial Guinea” OR Eritrea* OR “Eswatini” OR Swaziland OR Ethiopia* OR Gabon* OR Gambia* OR Ghana* OR Guinea OR “Guinea Bissau” OR Kenya* OR Lesotho OR Liberia* OR Libya* OR Madagascar OR Malawi* OR Mali OR Mauritania* OR Mauritius OR Morocco* OR Mozambique OR Namibia* OR Niger OR Nigeria* OR Rwanda* OR “Sao Tome and Principe” OR Senegal* OR Seychelle* OR “Sierra Leone” OR Somali* OR “South African” OR “South Africa” OR “South African” OR Sudan* OR “North Sudan” OR “South Sudan” OR Tanzania* OR Togo* OR Tunisia* OR Uganda* OR Zambia* OR Zimbabwe*). Citation lists of retrieved articles were manually screened to ensure sensitivity of the search strategy.

### Study selection

Studies were included if they (i) Provided a quantitative measure of disease occurrence (prevalence, incidence) or mortality (survival, mortality rate), and (ii) Had a quantitative association between risk factors and CCA. Studies were excluded if any of these criteria were not met. Titles and abstracts of all articles identified were screened independently by the authors. Full texts were reviewed by the authors, and consensus reached on potential eligibility.

### Data extraction

The following information was extracted from each eligible study from the database search: first author; year of publication; country of study; study population; study design; patient characteristics; management and outcomes.

### Quality assessment

A modified quality assessment of the final papers included in this systematic review was adapted from the quality assessment tool for systematic reviews of observational studies (QATSO) [22].

Items included in the QATSO tool were: (1) External validity (representativeness of sampling procedures used in each study); (2) Response rate, which we modified to include three categories (> 80%, 60%-80%, < 60% or not reported); (3) Validity of measurement methods and bias in the measurement of the outcomes; and (4) Control for important confounders. The final score was the mean across the items. Studies achieving a final mean score of 60% or above were considered adequate in this review [23].

### Statistical analysis

Statistical analyses were performed with Stata 15.1 (Stata Corporation, College Station, TX). Descriptive data were expressed as numbers with proportions. We used the χ2 test to compare proportions. *P* values < 0.05 were considered significant.

## Results

### Results of the search strategy

**Fig. 1:**
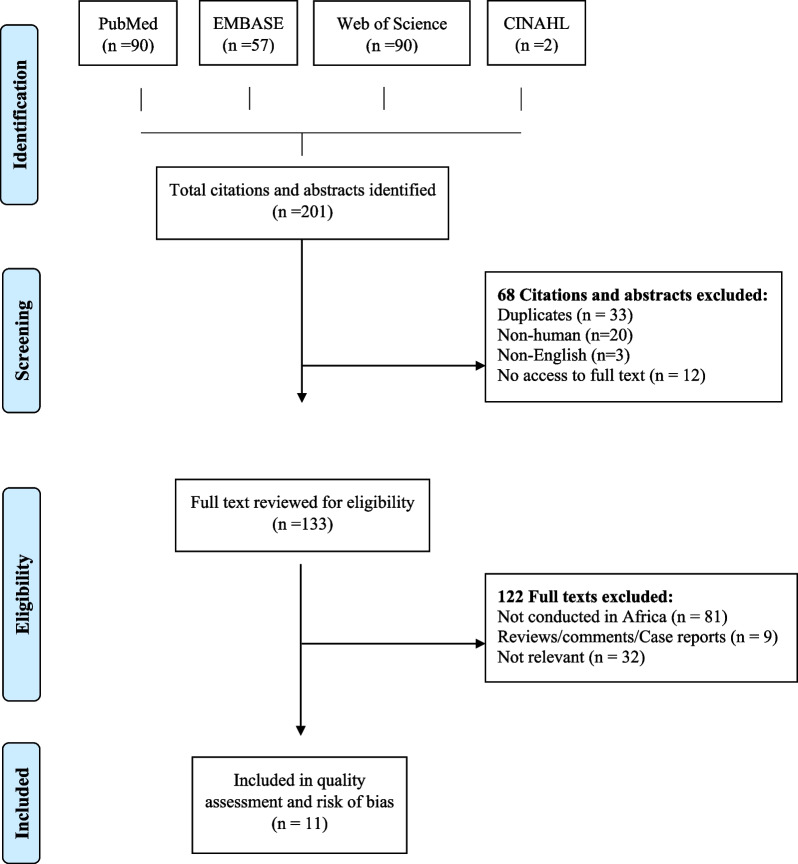
Flow diagram of included and excluded studies

A total of 201 citations were identified from the four databases. After excluding duplicates (n = 33), non-human citations (n = 20), non-English citations (n = 3), and articles that could not be accessed (n = 12), 133 full texts were reviewed for eligibility, and 11 studies were included (Fig. 1).

### Description of the studies and CCA patients

Of the 11 studies, one reported on the epidemiology and risk factors for CCA and was conducted in Nigeria, [24] and 10 studies reported on management and outcomes of CCA. These 10 studies also reported on patient characteristics that were included in the epidemiology and risk factor analysis below. Of these 10 studies, two studies were conducted in Tunisia, [25, 26] two in South Africa [27, 28], and six in Egypt (Table 1) [29–34].

**Table 1:** Summary of studies from systematic review

Number of studies	Nigeria	South Africa	Tunisia	Egypt
Per country	1	2	2	6
Reporting patient characteristics	1	1	2	4
Reporting on chemotherapy	–	–	2	1
Reporting transhepatic self-expanding metal stents	–	2	–	–
Reporting on preoperative biliary drainage	–	–	–	1
Reporting surgery		–	–	6

The 11 studies had a combined total of 1,125 patients with CCA. The study from Nigeria had 13 patients [24]. For the two studies from Tunisia, one had 10 patients [25] and the other had 28 patients [26]. For the two studies from South Africa, one had 36 patients [27]. And the other had 50 patients [28]. For the six studies from Egypt, one had 100 patients [29], two studies reported on the same 243 patients, [30, 34] one study had 440 patients [31], another had 46 patients [32], and lastly one study had 159 patients [33].

### Study quality

A qualitative assessment of these 11 studies achieved a final score of 77.3% and thus had adequate quality (Table 2).

**Table 2:** Quality assessment of the 11 studies included in the systematic review

Quality variable	Quality variable categories	Number of studies (%)
External validity	Non-probability	11/11 (100%)
	Probability	0
Response rate or proportion of patients managed for CCA	Above 80%	11/11 (100%)
	Between 60 and 80%	0
	Not reported	0
Validity of measurement methods and bias in the measurement of cholangiocarcinoma	Histology	7/11 (63.6%))
	Enhanced CT and/or MRCP	1
	Not reported	3
Control for important confounders	Yes	5/11 (45.6%)
	Not reported	6
Final mean % score (mean across the items)		77.3%

### Epidemiology and risk factors

The risk factors of CCA in Africa remain unclear, most occurring in absence of known risk factors. In a retrospective study done by Babatunde et al., in Nigeria, 13 histologically confirmed specimen of CCA were identified, of which 7 were from males (53.8%) [24]. The other studies that reported on gender in the patient characteristics also showed that more males than females had CCA [25, 27, 29, 30, 33, 34].

**Fig. 2:**
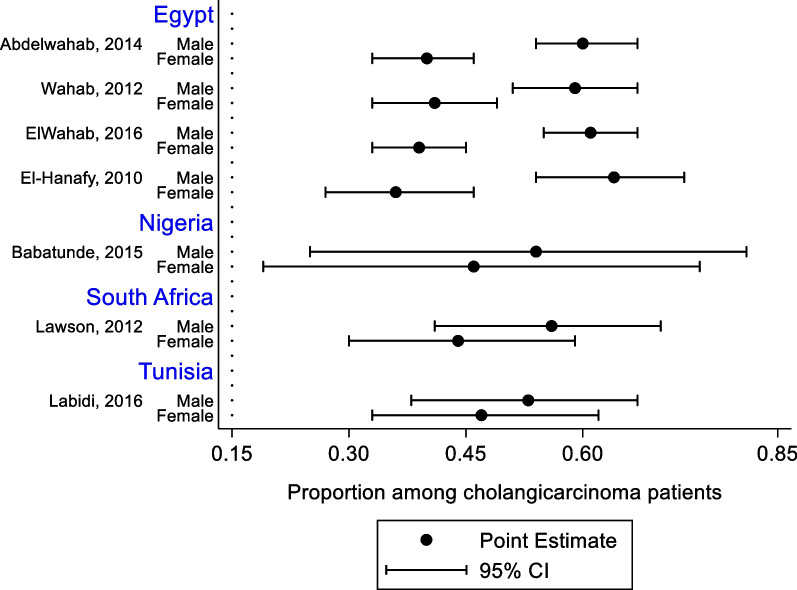
Proportion of males and females among patients with cholangiocarcinoma

The studies from Egypt showed that among patients with CCA, the proportion of males was statistically significantly higher than females. As shown by the wide 95% confidence intervals, the studies from Nigeria, South Africa, and Tunisia had small sample sizes of patients with CCA, and therefore could not demonstrate the significant gender disparity that exists among patients with CCA (Fig. 2).

In studies that reported on age, the median age for CCA was between 52.5 and 61 years [25–27, 30, 33].

### Management and outcomes

#### Chemotherapy

Chemotherapy treatment was mainly used for palliative treatment and was reported in three studies [25, 26, 32]. The chemotherapy was also administered as either neoadjuvant or adjuvant therapy [26]. In most cases, the first line chemotherapy used was gemcitabine based [25, 26]. The combined chemotherapies used include gemcitabine-oxaliplatin (GEMOX), gemcitabine-cisplatin (GEMCIS), and folfirinox (5-fluorouracil, leucovorin, irinotecan, and oxaliplatin) regimes. The second line chemotherapy used were capecitabine, irinotecan and paclitaxel. Adjuvant chemotherapy was mainly gemcitabine based [26].

#### Percutaneous transhepatic self-expanding metal stents (SEMS)

Only two studies reported the use of percutaneous transhepatic SEMS for palliation of malignant biliary obstruction [27, 28] In these patients no surgery was planned as the tumor was beyond the surgical margins. Lawson et.al reported that 40% of the SEMS was placed in a single stage procedure while the rest was a two-stage procedure. The biliary obstruction relief and SEMS placement procedure was successful in all the patients. The type of SEMS used was a Boston Scientific 69 mm × 10 mm Wall stent [28].

#### Preoperative biliary drainage (PBD)

This is a preoperative procedure done to relieve the patient from the jaundice and improve the patient’s general and liver functional state before any definitive surgery. The type of PBD reported in a single study includes percutaneous transhepatic biliary drainage (PTBD), and endoscopic retrograde cholangiopancreatography (ERCP) with stent placement [29].

#### Surgery

This is the only known curative treatment for cholangiocarcinoma. There were six studies in which patients underwent surgery for curative intent [29–34]. In each of these studies the choice of the surgical procedure done was dependent on the patients’ general status and tumor extension. Major hepatectomy with or without caudate lobectomy was reported in two studies [32, 33]. In all these studies, extrahepatic biliary resection and lymphadenectomy of locoregional lymph nodes was done. For patients with extrahepatic CCA which was localized, local resection of the bile duct with or without minor hepatectomy was performed [32]. Hepaticojejunostomy with or without a stent was done for biliary enteric anastomosis [33]. In one case–control study that compared patients that had both CCA and cirrhosis, with those that had CCA but without cirrhosis, the incidence of early postoperative liver cell failure was significantly higher in the cirrhotic group. Also, cirrhosis was associated with significantly lower overall survival [34]. However, not all surgery was for curative intent, in some cases palliative bypass surgery was done to relieve patient from jaundice secondary to malignant obstruction [27].

#### Assessment of outcomes and overall survival

The best median patient survival time reported was 36 months in patients who underwent surgery with curative intent. This was in patients that underwent major hepatectomy with caudate lobectomy [33]. Patients who underwent palliative bypass surgery had a reported survival rate of 54.5% in one year and the longest survival period of 28 months [27]. Two other studies with sample sizes of 10 and 28 CCA patients reported a median survival period of 10 months and 12 months, respectively [25, 26]. A single center experience study with a study population of 100 patients showed a higher median survival period of 19.7 months in patient who did not undergo pre-operative biliary drainage compared to those who underwent it prior to hepatectomy [29].

Palliative chemotherapy was used in patients whose CCA was beyond the surgical margins. Two studies on palliative chemotherapy with a combined study population of 68 patients had the same median survival period of 9 months [25, 26]. Also, two studies on palliative treatment of malignant biliary obstruction using a percutaneous transhepatic self-expanding metal stents had a median survival period of seven months and a 20% survival rate in six months [27, 28].

## Discussion

This is the first systematic review to assess risk factors, epidemiology, and management of cholangiocarcinoma in Africa. We have not identified a comprehensive study on the incidence of CCA across the whole continent of Africa. An Egyptian study has identified male gender (1.7:1), farming and rural residency, cirrhosis, hepatitis C infection (54%), Schistosomiasis (66.5%), chronic typhoid infection (52%) and gallstone disease as possible risk factors for hilar cholangiocarcinoma [35]. However, in a retrospective study done by Babatunde et al. in Nigeria, 37 patients had biliary tract carcinomas (representing 0.18% of all cancers in Ibadan), with more female than male patients (26 versus 11) [24]. Twenty females and four males had gallbladder carcinoma, while 6 females and 7 males had cholangiocarcinoma (p = 0.02). Gallstones (33%) and dysplasia (42%) were also risk factors for developing biliary type cancers [24].

CCAs express different types of mucin as a marker of differentiation and probable metastatic potential. MUC1, MUC1 core, MUC2, MUC3, MUC4, MUC5AC, and MUC6, were studied [36]. Extensive MUC3 expression was significantly associated with well-differentiated tumors, while there was an approaching significance between the extensive expression of MUC1 and metastasis in CCA [36]. Depot‐medroxyprogesterone acetate (DMPA) is not a risk factor for the development of either hepatocellular carcinoma or cholangiocarcinoma according to a study conducted in Thailand and Kenya [37]. PSC, a major risk factor in the west, is rare especially in sub-Saharan Africa as is ulcerative colitis. A study on PSC has been conducted in South Africa, with a total of 69 patients attending Charlotte Maxeke Johannesburg Academic Hospital of which 22 were black [38]. The risk of CCA among this population in Johannesburg has not been stated. 3 out of 4 Afro Caribbean women on the UC database at St Bartholomew’s Hospital in London developed of PSC [39]. Segal reported a series of the first 46 patients treated at Baragwanath Hospital with ulcerative colitis which is a known risk factor for PSC [40].

We have not seen any published evidence of risk of PSC and CCA in Africa. Fish borne zoonotic liver flukes *Clonorchis sinensis* and *Opisthorchis viverrini* are not a problem in Africa. There is a case report of infestation among Egyptian family who had the practice of consumption of imported fish from the Far East [41]. Morsy et al. describe liver flukes in an Egyptian family, however this is a case report and describes an infestation among the Egyptian family who had the practice of consumption of imported fish from the Far East and not a larger Africa population [41]. Whereas, this is not a risk factor among native Africans, it will be an increasing risk with Chinese migration onto the continent of Africa. It is estimated up to 12.5 million Chinese are infected by *Clonorchis sinensis* [42].

Other liver flukes causing fasciolasis affect cows and sheep in almost all countries in Africa [43]. Although there is a similar lifecycle and pathogenesis with *Clonorchis sinensis* and *Opisthorchis viverrini*, there is not definite causal effect for CCA. Gall stones have been identified as a risk factor for CCA. However, no direct cause link has been attributed to hemolytic anemias which causes pigment stones. Recent and innovative studies have explored the role of organoids as models for studying cholangiocarcinoma [45]. Sato et al. describe the use of 3D cell culture models to create an environment that contributes towards the understanding cholangiopathies including cholangiocarcinoma [45]. Wang et al. also delineate the impact of the microenvironment in liver carcinogenesis [46]. Exploring the role of microenvironment and developing models for the study of cholangiocarcinoma are channels to explore in understanding the epidemiology and risk factors [46].

Eleven studies report on the management of CCA in Africa, 6 in Egypt, 2 in South Africa, 2 in Tunisia and 1 in Senegal. Although liver transplantation is emerging as treatment of choice in localized hilar CCA (hCCA), this has not been reported in Africa. Six Egyptian and 2 Tunisian studies have reported on surgical resection of CCA with curative intent. In Egypt, Wahab reported that major hepatectomy with excision of the extrahepatic bile duct system and caudate lobe resection may be recommended for the surgical treatment of central cholangiocarcinoma in selected cases [32].

In a study published in 2012 Wahab concluded that caudate lobe resection in combination with major hepatectomy did not affect operative or postoperative morbidity and mortality but led to higher rates of margin-negative resections and significantly improved survival [33].

El- Hanafy et al. found that preoperative biliary drainage by PTC and ERCP in selected patients with cholangitis and long-standing jaundice increased morbidity, transfusion requirements and hospital stay [29]. However biliary drainage was associated with better outcomes in patients with malnutrition and renal impairment prior to liver resection in hCCA. But in these patients, there was higher complication risk including bile leak and collections, increased transfusion requirement, wound infection and pneumonia. In another study El Wahab et al. treated 243 patients with hCCA with resection of which 173 were with curative intent [30].

There was a 14% five-year survival. Factors influencing survival were young age at diagnosis, resection with caudate lobe resection, well differentiated tumor, negative resection margins, negative nodal metastases, and absence of cirrhosis. A bilirubin of less than 10 mg/dL and HCV negative status in a non-cirrhotic liver predicted a better prognosis in resection on hCCA [44].

Of the two Tunisian studies, the treatments were multimodal with different tumor locations. Romdhane et al., [25] treated 17 patients: 41% gall bladder, 35.5% pHCCA, 23.5% dCCA. Five patients were treated with curative intent, of which 3 had adjuvant chemotherapy (the subtype of CCA is not described), with rest treated with chemotherapy. Median survival for surgical resection was 10 months and 9 months for the chemotherapy group. Labidi S et al. also reports of treatment outcomes of 51 patients in Tunisia: 45% gall bladder, 22% hCCA, 20% iCCA 14.5% dCCA [26]. Of these, 9 were treated with curative resection 5 of whom also had adjuvant chemotherapy (subtype unclear). Again, the outcome was 12 months median survival for surgical resection with curative intent group, and 9 months in the chemotherapy only group.

Two South African studies report on palliative management of obstructing hCCA [27, 28], Clarke DL et al. report on a total of 36 deeply jaundiced patients with hilar obstruction [27]. Twenty-two had surgical biliary bypass, and 14 had PTC, the surgical group had higher morbidity, but both had good symptomatic relief of jaundice. There was no significant benefit of survival in the 2 groups, concluding that PTC would be treatment of choice in this group of patients. Lawson AJ et al. evaluated the use of PTC self-expanding metal stents to palliate malignant biliary obstruction as an alternative to surgical bypass or when ERCP is not feasible [28]. This study involved 50 patients. Although the mortality rate was high in this very high-risk group of patients, PTC placed SEMS achieved satisfactory palliation [28].

While the risk factors exist across the continent, it is unlikely that these data can be extrapolated across the whole of Africa. The changing demographics on the African continent with changing migration from the Far East should increase the awareness of *Clonorchis sinensis* and *Opisthorchis viverrini* as risk factors for CCA. The prevalence of PSC in Africa is unknown, as it is the only condition that warrants surveillance elsewhere in the world. Research is also required to determine if *Fasciola.hepatica* and *Fasciola.gigantica* are indeed not a risk factor for CCA, as they have a similar life cycle and cause cholestatic disease as do *Clonorchis sinensis* and *Opisthorchis viverrini.*

CCA remains a late presenting disease in Africa. The best median survival outcome of 36 months were for patients who had undergone hepatectomy with caudate lobectomy. Outcomes were improved when patients *did not* undergo preoperative biliary drainage. Where surgical resection with curative intent was planned, the data does not show that was based on modern radiological staging techniques and preoperative histology, and this may explain the poor 5-year survival. In a single high-volume center in Egypt, curative surgery results in 14% 5-year survival. At this center, factors positively influencing survival were identified as: caudate lobe resection, bilirubin of less than 10 mg/dL, absence of cirrhosis and young age. There are no obvious risk factors on which to formulate surveillance programs in Africa. PTC based SEMS can be used to palliate jaundice in obstructing tumors as can ERCP stenting of dCCA. This would require investment in expertise and equipment in those African countries lacking such equipment. Chemotherapy was used for palliative intent in most studies, except a select group from the Tunisian studies when it was neoadjuvant to curative resection [25, 26].

The earlier studies were Gemcitabine based, either with Oxaliplatin (GEMOX) or Cisplatin (GEMCIS). The Folfirinox (5-fluorouracil, leucovorin, irinotecan, and oxaliplatin) regime has also been used. The numbers in these study groups were small and there was not significant survival advantage in those who received chemotherapy and neoadjuvant chemotherapy.

Our Study limitations include the few numbers of included studies; inability to rule duplicate publication as similar author’s group published a few of the included studies and the data reviewed is reported from 4 countries only. Given the small number of studies, our review highlights the need for more research to understand epidemiology and aid with development of management strategies.

## Conclusion

Our systematic review contributes towards addressing the questions of epidemiology, etiology, risk factors, clinically outcomes and management of cholangiocarcinoma in Africa. Our current review provides some foundational published work needed to guide future studies and support proposed research. Eleven studies from four countries are not representative of the African continent hence the importance of this publication. There is limited data from the African continent differentiating pathogenesis of intraphepatic versus extrahepatic cholangiocarcinoma. The clinical presentation of patients in Africa is similar as in patients in other continents with extrahepatic cholangiocarcinoma, with the biliary obstruction as the most likely presentation. Further studies are required to explore the etiology, pathogenesis, management, and clinical outcomes of intra versus extrahepatic cholangiocarcinoma in Africa.

## Data Availability

All data generated and analysed is included in this publication.

## Abbreviations

CCA: Cholangiocarcinoma
PSC: Primary sclerosing cholangitis
MRCP: Magnetic resonance cholangiopancreatography
CA19-9: Carbohydrate antigen 19–9
iCCA: Intrahepatic cholangiocarcinoma
pCCA: Perihilar cholangiocarcinoma
dCCA: Distal cholangiocarcinoma
G6PD: Glucose-6-phosphate dehydrogenase
GLP-1: Glucagon-like peptide-1
ERCP: Endoscopic retrograde cholangiopancreatography
PTC: Percutaneous transhepatic cholangiography
QATSO: Quality assessment tool for systematic reviews of observational studies
GEMOX: Gemcitabine-oxaliplatin
GEMCIS: Gemcitabine-cisplatin
SEMS: Self-expanding metal stents
PBD: Preoperative biliary drainage
PTBD: Percutaneous transhepatic biliary drainage
hilar CCA: Hilar cholangiocarcinoma
DMPA: Depot‐medroxyprogesterone acetate
UC: Ulcerative colitis
HCV: Hepatitis C virus
